# Differences in motor inhibition in young and older musicians and non-musicians at rest

**DOI:** 10.3389/fnagi.2023.1230865

**Published:** 2023-09-08

**Authors:** Patricia Izbicki, Tessa Mendoza, Andrew Zaman, Elizabeth L. Stegemöller

**Affiliations:** ^1^Department of Kinesiology, Iowa State University, Ames, IA, United States; ^2^Hussman Institute for Human Genomics, Miller School of Medicine, University of Miami, Miami, FL, United States

**Keywords:** aging, motor inhibition, inhibitory control, music training, musicians

## Abstract

**Introduction:**

Older adults experience a decline in motor inhibition. These declines have been implicated in instrumental activities of daily living. However, studies have revealed that older musicians have behavioral and neurophysiological enhancements in various motor domains compared to non-musicians. This suggests that music training may delay the decline in motor inhibition with aging. Nevertheless, motor inhibition has not been studied in young or older musicians and non-musicians. Thus, the present study aimed to investigate the neurophysiological differences in motor inhibition in aging musicians and non-musicians.

**Methods:**

A total of 19 healthy young adult musicians, 16 healthy young non-musicians, 13 healthy older adult musicians, and 16 healthy older adult non-musicians were recruited for the study. Transcranial magnetic stimulation single-pulse (SP) and short interval cortical inhibition (SICI) were performed at rest and then converted into inhibition percentage.

**Results:**

We did not observe significant differences between young and older musicians and non-musicians in resting SP MEP. Older adults had lower resting SICI MEP than young adults. Older adults (36%) had a greater percentage of inhibition than young adults (16%). However, when controlling for background EMG activity, musicians had a lower inhibition percentage than non-musicians.

**Discussion:**

The results revealed that, despite the greater use of spinal mechanisms, decreased SICI, and increased inhibition percentage in older adults, motor inhibitory circuitry remains intact and functional in both young and older musicians and non-musicians. Future studies will reveal whether there are differences in motor inhibition during movement in musicians across a person's lifespan.

## Introduction

With a projected increase of more than 2 billion in the population of older adults over the age of 60 years in 2050 (DESA UN., 2013), there is a pressing need to develop effective interventions to improve the quality of life and promote active engagement for older adults. One method is to target strategies that improve motor inhibition (i.e., the suppression of unwanted movement) (Nigg, 2000; MacLeod, 2007; Tiego et al., 2018). A decrease in inhibitory control in aging (Nielson et al., 2002; van Hooren et al., 2007; Heise et al., 2013; Wolf et al., 2014) leads to the loss of the ability to complete activities of daily living as well as diminished quality of life (Royall et al., 2004; Jefferson et al., 2006). Particularly, deficits in motor inhibition due to aging may lead to longer reaction times, impaired coordination skills, and reduced fine motor functions (Levin et al., 2014).

Neurophysiological changes support the observed behavioral motor inhibition deficits. These changes include (1) a reduction in gray matter brain volume, (2) a reduction in white matter volume, and/or (3) biochemical changes in the brain (Seidler et al., 2010; Hu et al., 2014; Fujiyama et al., 2016; Fernandez-Ruiz et al., 2020). Decreased integrity of white matter tracts in the fronto-basal-ganglia network has been associated with decreased motor inhibition in older adults (Coxon et al., 2012). Interhemispheric and intrahemispheric inhibition in the brain while completing motor tasks has also been shown to decline during healthy aging (Ruitenberg et al., 2019). Furthermore, lower gamma-aminobutyric acid (GABA) levels in the pre-supplementary motor area were associated with poorer motor inhibition using the stop-signal task (Hermans et al., 2018; Pauwels et al., 2018). In short, motor inhibition is impaired in older individuals. However, there remains a paucity of research examining potential strategies to improve motor inhibition in older adults.

Music training may be a viable option and has been shown to alter healthy young adult motor inhibitory performance and neurophysiology (Hughes and Franz, 2007; Rosenkranz et al., 2007; Penhune, 2011). Slater et al. (2018) showed increased motor inhibition and decreased variability in motor inhibition in musicians (specifically drummers) during a rhythmic task. Nevertheless, little research has examined the effects of music training on older adults. Only one experiment has examined differences in motor inhibition between older musicians and non-musicians. Moussard et al. (2016) demonstrated differences in brain activity in older adult musicians compared to non-musicians during motor inhibition using the go/nogo task. While these results are promising, there remains a critical need to address the behavior and neural correlates of motor inhibition in older adult musicians and non-musicians.

The use of transcranial magnetic stimulation (TMS) has been shown to be an effective tool for understanding changes in motor cortical activity in older adults. Oliviero et al. (2006) showed that single-pulse motor evoked potential (MEP) (i.e., hand twitch) amplitudes at rest are reduced in older adults compared to young adults, indicating a reduction in motor cortical excitability, possibly due to the loss of cortical and spinal motor neurons in older adults. Research measuring short-interval intracortical inhibition (SICI), which arises from axonal refractoriness and low-threshold GABA_a_ receptor-mediated inhibition (Vahabzadeh-Hagh et al., 2011) during periods of rest, has revealed that reduced SICI (i.e., reduced inhibition) is associated with poorer performance in motor inhibition in older adults (Peinemann et al., 2001; McGinley et al., 2010; Heise et al., 2013). Reduced SICI in older adults has also been found in studies measuring SICI during movement (Fujiyama et al., 2011; Heise et al., 2013). Taken together, these results demonstrate that TMS is a valid method for examining differences in aging musicians.

Using TMS, Nordstrom and Butler (2002) showed reduced intracortical inhibition of corticospinal neurons in musicians. Rosenkranz et al. (2007) used SICI to show an increase in motor inhibition in musicians compared to non-musicians. Vaalto et al. (2016) showed that musicians specifically display more motor inhibition in the non-primary areas of the motor cortex than non-musicians. Musicians also showed that proprioceptive stimuli exerted stronger inhibition effects on corticospinal excitability, suggesting greater motor inhibition for specific somatosensory inputs. Márquez et al. (2018) showed using single-pulse TMS that musicians exhibit greater motor cortical inhibition during the preparation of isolated and complex finger movements. Furthermore, musicians have shown greater interhemispheric inhibition during finger movement (2016). In short, various studies using single-pulse and SICI TMS demonstrate differences in motor inhibition between musicians and non-musicians.

To summarize, the neurophysiology behind motor inhibition in musicians involves reduced motor cortical activity and increased inhibition in motor corticospinal circuits. Unfortunately, no studies have revealed neurophysiological differences in motor inhibition in young and older musicians and non-musicians. Thus, this study aimed to determine the differences in neurophysiological measures of motor inhibition at rest in older and young adult musicians and non-musicians. We hypothesize that (1) musicians will demonstrate decreased MEP amplitude compared to non-musicians (i.e., increased inhibition percentage) and (2) young adult musicians and non-musicians will demonstrate decreased MEP amplitude (i.e., increased inhibition percentage) compared to older adult musicians and non-musicians.

## Methods

### Participants

All participants provided written informed consent to participate in the study, which was approved by the Iowa State University Institutional Review Board. All procedures involving human participants were in accordance with the institution's ethical standards and with the 1964 Helsinki Declaration and its later amendments or comparable ethical standards.

The inclusion criteria for all young and older adults included (1) those aged between 18 and 35 and between 65 and 80 years, (2) instrumental musicians (defined as currently practicing) or non-musicians, and (3) no neurological, musculoskeletal, and/or neuropsychiatric disorder. The exclusion criteria included (1) significant cognitive impairment (Mini-Mental State Exam < 24), (2) major depression (Beck Depression Inventory > 18), any previous adverse reactions to TMS, previous seizure, surgery on blood vessels, the brain, or the heart, previous stroke, severe vision or hearing loss, metal in the head, implanted devices, severe headaches, previous brain-related conditions, brain injury, medications (i.e., antibiotics, antifungal, antiviral, antidepressants, antipsychotics, chemotherapy, amphetamines, bronchodilators, anticholinergics, antihistamines, and sympathomimetics), family history of epilepsy, pregnancy, alcohol consumption less than 24 h before the study, smoking, and illicit drug use.

A total of 19 healthy young adult (HYA) musicians, 16 HYA non-musicians, 13 healthy older adult (HOA) musicians, and 16 HOA non-musicians were recruited for the study. Demographic data collected included age, gender, ethnicity, education, handedness, marital status, yearly income, and hours of physical activity (Table 1). The Lubben Social Network Score was collected to control for variability in social engagement among participants (Lubben et al., 2006). Shipley-2 (IQ assessment) was collected to control for existing variability in cognitive function and ability (Kaya et al., 2012). Gordon's Advanced Measures of Music Audiation (AMMA) were collected to control for existing variability in music aptitude (Gordon, 1989). The Musical Experience Questionnaire (MEQ) was collected to assess years of music experience, years of formal training, and current practice hours (Bailey and Penhune, 2012).

**Table 1:** Healthy young adults (HYA) and healthy older adult (HOA) demographic information.

**Demographics**	**HYA musicians**	**HYA non-musicians**	**HOA musicians**	**HOA non-musicians**
Gender (% women, % men)	32%, 68%	63%, 37%	62%, 38%	38%, 63%
Age (Mean ± SD)	20 (± 3)	23 (± 5)	68 (± 4)	71 (± 4)
Handedness (% RH, % LH)	89%, 11%	94%, 6%	92%, 8%	100%, 0%
Ethnicity (% Caucasian, % Asian, % Latino, % Mixed, % African American)	68%, 21%, 0%, 11%, 0%	55%, 13%, 13%, 0%, 19%	100%, 0%, 0%, 0%, 0%	100%, 0%, 0%, 0%, 0%
Physical activity/Week (Mean ± SD)	6 (± 4)	5 (± 3)	7 (± 7)	13 (± 13)
Highest education (% High School, % Bachelors, % Masters, % Professional)	79%, 21%, 0%, 0%	56%, 25%, 19%, 0%	0%, 47%, 15%, 38%	13%, 56%, 25%, 6%
Years of education (Mean ± SD)	14 (± 2)	16 (± 3)	19 (± 3)	17 (± 2)
GPA (Mean ± SD)	3.6 (± 0.4)	3.4 (± 0.6)	3.6 (± 0.4)	3.1 (± 0.5)
Marital status (% Single, % Married)	95%, 5%	100%, 0%	0%, 100%	13%, 87%
Yearly income (Dollars) (Mean ± SD)	4,684 (± 6,675)	14,575 (± 12,193)	85,730 (± 46,694)	66,625 (± 35,424)
Lubben social network score (Mean ± SD)	52 (± 13)	46 (± 12)	51 (± 12)	54 (± 11)
Shipley-2 vocabulary score (Mean ± SD)	32 (± 3)	32 (± 2)	36 (± 2)	33 (± 4)
Shipley-2 abstraction score (Mean ± SD)	18 (± 3)	16 (± 3)	17 (± 3)	16 (± 2)
Shipley-2 pattern score (Mean ± SD)	22 (± 4)	20 (± 4)	16 (± 5)	15 (± 5)
AMMA score (Mean ± SD)	59 (± 6)	46 (± 8)	57 (± 7)	50 (± 3)
Instrumental start age (Mean ± SD)	12 (± 4)	6 (± 6)	15 (± 16)	10 (± 5)
Number of years playing an instrument (Mean ± SD)	8 (± 4)	1 (± 2)	53 (± 16)	6 (± 9)
Current hours of practice/week (Mean ± SD)	7 (± 4)	N/A	8 (± 7)	N/A
Family music experience % Yes, % No)	74%, 26%	37%, 63%	85%, 15%	56%, 44%
Level of education (Mother) (% Middle School, % High School, % Bachelors, % Masters, % Professional)	0%, 32%, 53%, 10%, 5%	6%, 44%, 12%, 19%, 6%, 13%	8%, 78%, 7%, 7%, 0%	13%, 69%, 6%, 13%, 0%
Level of education (Father) (% Middle School, % High School, % Bachelors, % Masters, % Professional)	0%, 26%, 47%, 16%, 11%	13%, 25%, 25%, 19%, 6%, 12%	8%, 62%, 8%, 7%, 15%	19%, 63%, 12%, 0%, 6%

SD, standard deviation; GPA, grade point average. Shaded cells indicate a p-value of < 0.05 between groups.

### Data collection

For TMS, the motor hot spot, specifically the hand knob area in the primary motor cortex (M1), was located on the contralateral hemisphere of the dominant hand. The location and coil orientation (45 degrees to the left of the longitudinal fissure) were marked. For the data collected at rest, resting motor threshold (RMT) (i.e., an MEP at an amplitude of at least 50 μV produced for 5 out of 10 trials or 50% of the time) was then found (Rotenberg et al., 2014). RMT was completed in 20 min. Single-pulse TMS intensity was set at 120% RMT. The SICI conditioning pulse was set at 80% RMT, and the SICI test pulse was set at 120% RMT. The interstimulus interval was 3 ms.

Participants were seated in an armchair with their dominant forearm pronated and resting on the armrest. The participants were asked not to move during TMS. Single-pulse and SICI TMS were applied to the primary motor cortex dominant hand area using the Magstim Model 200 (Magstim, Whitland, and Carmarthenshire). The coil was a figure-8 coil (7 cm outer diameter of wings). The coil current was induced approximately perpendicular to the motor homunculus and central sulcus. The waveform was monophasic. Spike2 was used to trigger single-pulse and SICI stimulations via a Power 1401 data acquisition board and Spike2 software (Cambridge Electronic Design (CED), Cambridge, UK).

Motor-evoked potentials (MEPs) were recorded from the dominant first dorsal interosseous (FDI) using bipolar surface electromyography (EMG) (Delsys, Boston, MA, USA). Ten single-pulse stimulations and ten SICI stimulations per participant were applied during rest. Single-pulses and SICI stimulations were applied approximately every 5–12 s (for a total of 83–85 s in each condition).

### Data analysis

The primary neurophysiological outcome measure for motor inhibition was MEP amplitude. EMG signals were notch-filtered (60 Hz) and high-pass-filtered (second-order dual-pass Butterworth filter, 2 Hz cut-off). EMG signals were also DC-shifted, and the root mean square of the EMG signal was obtained. Peak-to-peak amplitude (μV) was obtained within 100 ms of the TMS pulse. To ensure that the MEP was not due to muscle tensing, background EMG was determined for periods of 1.25–0.25 s before the peak maximum amplitude and 0.25–1.25 s after the peak maximum amplitude. Background EMG trials > 10 μV were discarded (Majid et al., 2015). For EMG activity before peak amplitude, there were no trials discarded. For EMG activity after peak amplitude, there were no trials discarded. The raw data for each participant in the background EMG activity and each condition (i.e., single-pulse and SICI) was naturally log transformed to obtain a normal distribution. The primary outcome measure of MEP amplitude was obtained by averaging the natural log-transformed 10 MEP trials for each condition (i.e., single-pulse and SICI) (Nielsen, 1996; Clark et al., 2004; Izbicki et al., 2020). SICI was also expressed as a percentage using the formula: inhibition percentage (%) = 100 – [rest SICI MEP (conditioned pulse)/rest SP MEP (non-conditioned pulse) × 100] (Byblow and Stinear, 2006).

### Statistical analysis

Statistical analysis was completed in IBM SPSS Statistics for Windows, Version 25.0 (IBM Corp., Armonk, New York, USA). Normality was assessed using the Shapiro–Wilk test. Independent *t*-tests, the Mann–Whitney *U-*tests, and the Chi-squared tests were used to determine any differences in the demographics of young and older musicians and non-musicians. Tables 2, 3 demonstrate the types of statistical tests (i.e., the Chi-squared test, the independent *t*-test, and the Mann–Whitney *U-*test) completed on the demographic data based on categorical vs. continuous variables and the normality of the data. Significance was set to α = 0.05.

**Table 2:** Healthy young adult (HYA) musician and non-musician demographic statistical tests and results.

**Demographics**	**Statistical test**	**Result**
Gender	Chi-squared test	χ(1) = 3.35, *p* = 0.067
Age	Mann–Whitney *U*-test	*U* = 77.0, *p* = 0.012
Handedness	Chi-squared test	χ(1) = 0.203, *p* = 0.653
Ethnicity	Chi-squared test	χ(4) = 8.20, *p* = 0.085
Physical activity/week	Mann–Whitney *U*-test	*U* = 138.5, *p* = 0.652
Highest education	Chi-squared test	χ(2) = 4.27, *p* = 0.118
Years of education	Mann–Whitney *U*-test	*U* = 84.5, *p* = 0.023
GPA	Mann–Whitney *U*-test	*U* = 131.0, *p* = 0.486
Marital status	Chi-squared test	χ(1) = 0.867, *p* = 0.352
Yearly income	Mann–Whitney *U*-test	*U* = 77.0, *p* = 0.012
Lubben Social Network score	Independent *T*-test	*t*(33) = 1.38, *p* = 0.178
Shipley-2 vocabulary score	Independent *T*-test	*t*(33) = −0.56, *p* = 0.583
Shipley-2 abstraction score	Independent *T*-test	*t*(33) =1.51, *p* = 0.140
Shipley-2 pattern score	Mann–Whitney *U*-test	*U* = 108.5, *p* = 0.147
AMMA score	Independent *T*-test	*t*(33) = 5.43, *p* < 0.001
Instrumental start age	Mann–Whitney *U*-test	*U* = 70.0, *p* = 0.006
Number of years playing an instrument	Mann–Whitney *U*-test	*U* = 20.0, *p* = < 0.001
Family music experience	Chi-squared test	χ(1) = 4.64, *p* = 0.031
Level of education (Mother)	Chi-squared test	χ(5) = 7.31, *p* = 0.20
Level of education (Father)	Chi-squared test	χ(5) = 9.93, *p* = 0.077

GPA, grade point average; AMMA, Advanced Measures of Music Audiation. Shaded cells indicate a p-value of < 0.05 between groups.

**Table 3:** Healthy young older (HOA) musician and non-musician demographic statistical tests and results.

**Demographics**	**Statistical test**	**Result**
Gender	Chi-squared test	χ(1) = 1.66, *p* = 0.198
Age	Independent *t*-test	*t*(27) = −1.93, *p* = 0.064
Handedness	Chi-squared test	χ(1) = 1.28, *p* = 0.259
Ethnicity	Chi-squared test	N/A
Physical activity/week	Mann–Whitney *U*-test	*U* = 71.5, *p* = 0.152
Highest education	Chi-squared test	χ(3) = 5.68, *p* = 0.128
Years of education	Mann–Whitney *U*-test	*U* = 61.0, *p* = 0.055
GPA	Independent *T*-test	*t*(27) = 2.69, *p* = 0.012
Marital status	Chi-squared test	χ(1) = 1.75, *p* = 0.186
Yearly income	Independent *T*-test	*t*(27) = 1.25, *p* = 0.221
Lubben Social Network score	Independent *T*-test	*t*(27) = −0.60, *p* = 0.554
Shipley-2 vocabulary score	Mann–Whitney *U*-test	*U* = 42.0, *p* = 0.006
Shipley-2 abstraction score	Independent *T*-test	*t*(27) = 0.787, *p* = 0.438
Shipley-2 pattern score	Independent *T*-test	*t*(27) = 0.761, *p* = 0.454
AMMA score	Independent *T*-test	*t*(27) = 3.41, *p* = 0.002
Instrumental start age	Mann–Whitney *U*-test	*U* = 103.0, *p* = 0.965
Number of years of playing an instrument	Mann–Whitney *U*-test	*U* = 10.0, *p* < 0.001
Family music experience	Chi-squared test	χ(1) = 5.09, *p* = 0.024
Level of education (Mother)	Chi-squared test	χ(3) = 0.408, *p* = 0.94
Level of education (Father)	Chi-squared test	χ(4) = 2.61, *p* = 0.626

GPA, grade point average; AMMA, Advanced Measures of Music Audiation. Shaded cells indicate p < 0.05 between groups.

As stated in the previous section, EMG activity before and after peak amplitudes and single-pulse and SICI MEP were log-transformed. Although the data were not normally distributed for EMG activity before and after peak amplitude for both single-pulse and SICI conditions and peak-to-peak single-pulse MEP post-transformation, Blanca et al. (2017) showed that an ANOVA is a valid option with non-normal data. Thus, to confirm that potential differences in MEP amplitude are due to cortical mechanisms rather than an increase in drive to spinal mechanisms, a 2 (young adult, older adult) × 2 (musician, non-musician) ANOVA was conducted to compare 1.25–0.25 s before the peak maximum amplitude among the three conditions as well as 0.25–1.25 s after the peak maximum amplitude among the three conditions. To examine differences in motor inhibition at rest (i.e., peak-to-peak amplitude and inhibition percentage of the MEP), a 2 (young adult, older adult) × 2 (musician, non-musician) ANOVA was completed. Bonferroni correction was used for *post-hoc* analysis (HYA musicians vs. HYA non-musicians, HOA musicians vs. HOA non-musicians). Significance was set at α = 0.025.

## Results

### Participants

The young adults did not differ in gender, handedness, ethnicity, physical activity per week, highest education, GPA, or marital status (Tables 1, 2). The following also did not differ: social engagement, assessed using the Lubben Social Network scale (*p* = 0.18), cognitive function and ability, evaluated using the Shipley-2 vocabulary, abstraction, and pattern scores (*p* = 0.58, *p* = 0.14, *p* = 0.13), and parental education (both mother and father), measured using the MEQ (*p* = 0.22, *p* = 0.25). The young adults differed in age, education years, and yearly income (Tables 1, 2). They also differed in musical aptitude as measured by the AMMA (*p* < 0.001), instrumental start age (*p* < 0.006), and number of years playing an instrument (*p* < 0.001). Family musical experience also differed between groups (*p* = 0.03). HYA musicians demonstrated a greater musical aptitude, a later instrumental start age, and a greater family musical experience. HYA musicians began playing at 12 years (± 4), while HYA non-musicians began playing at 6 years (± 6), which was statistically significant (*p* < 0.006). HYA musicians played their instruments for 8 years (± 4), while HYA non-musicians played their instruments for 1 year (± 2), which was statistically significant (*p* < 0.001) (Table 1). Moreover, 74% of HYA musicians had one or more immediate family members that played a musical instrument, while 37% of HYA non-musicians had one or more immediate family members that played a musical instrument (Table 2).

The older adults did not differ in gender, age, handedness, ethnicity, physical activity per week, years of education, marital status, or yearly income (Tables 1, 3). Social engagement, as measured using the Lubben Social Network scale (*p* = 0.55), cognitive function and ability, as measured via the Shipley-2 abstraction and pattern scores (*p* = 0.44, *p* = 0.45), instrumental start age (*p* = 0.97), and parental education (mother and father), as measured using the MEQ (*p* = 0.94; *p* = 0.63), did not differ. The older adults did differ in the highest education achieved and GPA (Tables 1, 3). Cognitive function and ability as measured via Shipley-2 vocabulary scores (*p* = 0.006), musical aptitude as measured by AMMA (*p* = 0.002), number of years of playing an instrument (*p* = 0.001), and family musical experience (*p* = 0.02) did differ. HOA musicians demonstrated a greater vocabulary performance, a musical aptitude, a greater number of years playing an instrument, and greater family musical experience. HOA musicians began playing at 15 (± 16) years, while HOA non-musicians began playing at 10 (± 5) years, which was not statistically significant (*p* = 0.97). HOA musicians played their instruments for 53 (± 16) years, while HOA non-musicians played their instruments for 6 (± 9) years, which was statistically significant (*p* = 0.001) (Table 3). Moreover, 85% of HOA musicians had one or more immediate family members that played a musical instrument, while only 56% of HOA non-musicians had one or more immediate family members that played a musical instrument.

### Pre- and post-background EMG

The results revealed no main effect of pre-background EMG activity for rest single-pulse in age [*F*_(1, 60)_ = 2.5, *p* = 0.12, = 0.041], group [*F*_(1, 60)_ = 0.95, *p* = 0.33, = 0.016], or age × group interaction [*F*_(1, 60)_ = 0.1.6, *p* = 0.21, = 0.026]. The results also revealed a main effect of age [*F*_(1, 60)_ = 15.8, *p* < 0.001, = 0.21] for pre-background EMG activity for rest SICI. Older adults had more pre-background EMG activity [1.4 ln(μV)] than young adults [0.82 ln(μV)]. There was no main effect of pre-background EMG for rest SICI for the group [*F*_(1, 60)_ = 0.46, *p* = 0.50, = 0.008] or age × group interaction [*F*_(1, 60)_ = 0.34, *p* = 0.57, = 0.006] (Table 4).

**Table 4:** Healthy young adult (HYA) and healthy older adult (HOA) pre- and post-background EMG.

**Pre-background EMG**				
	**HYA musicians**	**HYA non-musicians**	**HOA musicians**	**HOA non-musicians**
Rest single-pulse	1.07 (± 0.59)	1.03 (± 0.41)	1.12 (± 0.36)	1.42 (± 0.71)
Rest SICI	0.82 (± 0.24)	0.83 (± 0.48)	1.30 (± 0.70)	1.48 (± 0.77)
**Post-background EMG**				
	**HYA musicians**	**HYA non-musicians**	**HOA musicians**	**HOA Non-musicians**
Rest single-pulse	1.11 (± 0.67)	1.10 (± 0.44)	1.12 (± 0.36)	1.43 (± 0.72)
Rest SICI	0.86 (± 0.36)	0.83 (± 0.46)	1.57 (± 1.54)	1.28 (± 0.79)

Means and standard deviations [ln (μV)] shown. Shaded cells indicate a p-value of < 0.05 for the main effect of age.

The results revealed no main effect of post-background EMG activity for rest single-pulse in age [*F*_(1, 60)_ = 0.1.4, *p* = 0.24, = 0.023], group (*F*_(1, 60)_ = 1.2, *p* = 0.28, < 0.019], or age × group interaction [*F*_(1, 60)_ = 1.3, *p* = 0.27, = 0.020]. The results revealed a main effect of age [*F*_(1, 60)_ = 10.02, *p* = 0.002, = 0.14] for rest SICI. Older adults have greater post-background EMG activity [1.5 ln(μV)] than young adults [0.84 ln(μV)]. There was no main effect of post-background EMG activity for rest SICI in the group [*F*_(1, 60)_ = 0.087, *p* = 0.77, = 0.001] or age × group interaction [*F*_(1, 60)_ = 0.023, *p* = 0.88, = 0.0003] (Table 4).

### MEP peak to peak

#### Rest

**Figure 1:**
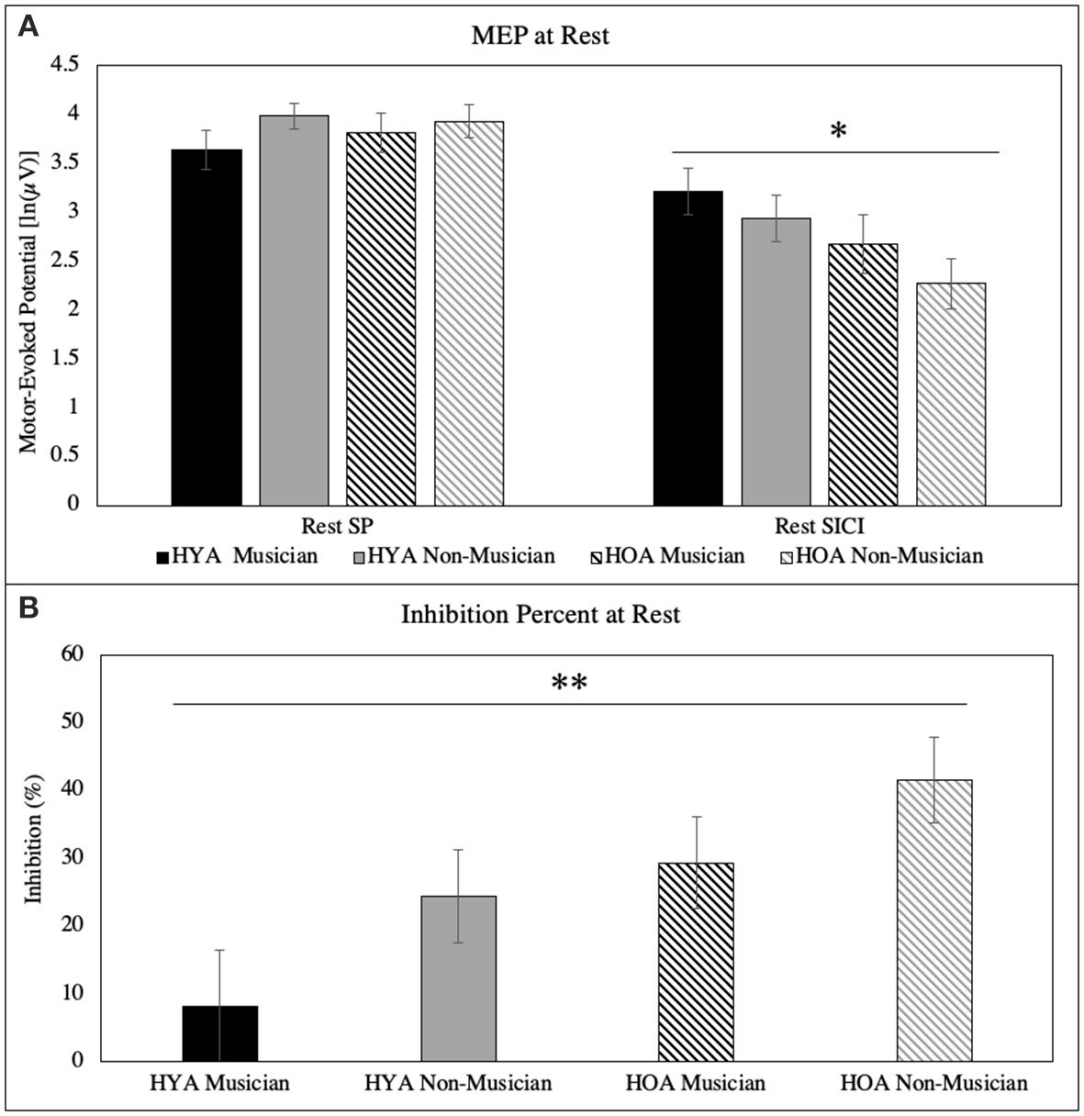
**(A)** Peak-to-peak MEP for single-pulse and SICI at rest. There was a main effect of age for SICI at rest. **(B)** Inhibition (%) at rest for each group. There was a main effect of age and group. All error bars reflect standard error *p = 0.02; **p = 0.01.

The results revealed no main effect of single-pulse MEP peak to peak in age [*F*_(1, 60)_ = 0.12, *p* = 0.74, = 0.028], group [*F*_(1, 60)_ = 1.7, *p* = 0.20, = 0.028], or age × group interaction [*F*_(1, 60)_ = 0.40, *p* = 0.53, < 0.007]. When background EMG activity (pre- and post-) were included as covariates, the results further revealed no main effects of single-pulse MEP peak to peak in age [*F*_(1, 58)_ = 0.26, *p* = 0.61, = 0.005], group [*F*_(1, 58)_ = 1.4, *p* = 0.25, = 0.023], or age × group interaction [*F*_(1, 58)_ = 0.36, *p* = 0.55, = 0.006] (Figure 1A).

The results revealed a main effect of SICI MEP peak to peak in age [*F*_(1, 60)_ = 5.4, *p* = 0.024, = 0.082]. Older adults have lower rest SICI MEP [2.5 ln(μV)] than young adults [3.1 ln(μV)]. There was no main effect of rest SICI MEP in the group [*F*_(1, 60)_ = 1.7, *p* = 0.20, = 0.028] or age × group interaction [*F*_(1, 60)_ = 0.065, *p* = 0.80, < 0.001]. When background EMG activity (pre and post) were included as covariates, the results revealed no main effects of SICI MEP peak to peak in age [*F*_(1, 58)_ = 1.8, *p* = 0.18, = 0.030], group [*F*_(1, 58)_ = 1.4, *p* = 0.24, = 0.024], or age × group interaction [*F*_(1, 58)_ = 0.030, *p* = 0.86, = 0.001] (Figure 1A).

### Inhibition percentage

The results revealed a main effect of inhibition percentage in age [*F*_(1, 60)_ = 6.7, *p* = 0.012, = 0.100]. Older adults (36%) have a greater percentage of inhibition at rest than young adults (16%). There was no main effect for group [*F*_(1, 60)_ = 3.7, *p* = 0.060, = 0.058] and age × group interaction [*F*_(1, 60)_ = 0.075, *p* = 0.79, = 0.001]. When background EMG activity (pre- and post-) were included as covariates, the results revealed a main effect of inhibition percentage in the group [*F*_(1, 58)_ = 4.3, *p* = 0.041, = 0.070]. Musicians had a lower percentage of inhibition at rest (17%) than non-musicians (33%). There was no main effect for age [*F*_(1, 58)_ = 2.9, *p* = 0.092, = 0.048], and age × group interaction [*F*_(1, 58)_ = 0.054, *p* = 0.82, = 0.001] (Figure 1B).

## Discussion

### MEP peak to peak single-pulse and SICI at rest

Although there were no differences in resting single-pulse MEP in our study, there are studies that support our results. Fathi et al. (2010) examined single-pulse MEP at rest before and after paired associative stimulation (PAS), a TMS technique that induces neuroplasticity. Although they showed differences in single-pulse MEP compared to young adults, it was only after PAS. Similar results were observed in the study by Freitas et al. (2011), in which there were no differences in single-pulse MEP for young, middle, and older participants before PAS was applied. In other words, there was no difference between young and older adults' single-pulse MEP before inducing neuroplasticity. Thus, it seems that excitatory motor circuitry itself is not affected by age but rather by neuroplasticity and functional connectivity. It also supports the notion that spinal mechanisms drive motor inhibition (discussed in the *Spinal Mechanisms via Background EMG* section below).

The age differences in SICI at rest are not surprising. Studies have shown that underlying motor inhibition measured using SICI differs between older and young adults (Peinemann et al., 2001; McGinley et al., 2010). An extensive body of literature suggests that SICI represents GABA_A_ inhibitory neurotransmission (Bhandari et al., 2016). GABA_A_ receptors are vulnerable to age-related deficits in GABAergic neurotransmission (Yu et al., 2006). Furthermore, animal studies have shown a decline in the total number of GABAergic neurons (Hua et al., 2008), alternations in GABA_A_ receptor subunit composition and function (Caspary et al., 1999; Yu et al., 2006; Schmidt et al., 2010), and the loss of the amount of GABA neurotransmitter (Ling et al., 2005) with aging. Our results indicate that, through the SICI paradigm, GABA_A_ receptor-mediated inhibitory neurotransmission seems to be intact in young and older adults at rest.

Although studies have shown that underlying motor inhibition measured by SICI is different between older and young adults (Peinemann et al., 2001; McGinley et al., 2010), there have been studies that are consistent with the non-significant results (or lack thereof) we obtained when controlling for background EMG activity. A recent meta-analysis showed that older adults demonstrated non-significant SICI differences compared to young adults (Bhandari et al., 2016). Other studies have also shown no significant differences (Smith et al., 1985; Rogasch et al., 2009; Cirillo et al., 2010; Opie and Semmler, 2014). Thus, it seems that the observed SICI age differences are potentially due to individual and methodological differences. However, it seems unlikely that our lack of results is due to our methodology alone because other studies using similar methodologies have not found any significant differences (Smith et al., 1985; Opie and Semmler, 2014). However, individual differences in our sample population, such as our older adults being both physically and cognitively active (see Table 1), may contribute to intact inhibitory neurotransmission at rest. Furthermore, spinal mechanisms (discussed in the next section) may be driving differences rather than cortical mechanisms.

Interestingly, when controlling for background EMG activity, musicians (regardless of age) showed a lower inhibition percentage than non-musicians. These results support previous literature showing that musicians' and non-musicians' brains appear to have differences in volume, morphology, density, connectivity, and function (Merrett et al., 2013). Rosenkranz et al. (2007) showed steeper recruitment of corticospinal excitatory and intracortical inhibitory projections in young musicians. However, the parameters of TMS stimulation and data analysis were different than in our paradigm. This could explain why we were not able to observe differences between musicians and non-musicians using single-pulse.

Furthermore, Hirano et al. (2020) found identical results to ours. They showed no differences in resting single pulses between musicians and non-musicians. In short, the motor cortical excitability and motor inhibitory circuitry appear to be intact at rest between aging musicians and non-musicians. However, studies have shown differences in single-pulse TMS in older adults (Opie and Semmler, 2014) and musicians (Clark et al., 2004) performing movements such as finger tapping. Future studies should examine single-pulse and SICI during movement to investigate whether this elicits cortical differences in aging musicians vs. non-musicians.

### Spinal mechanisms via background EMG

Background EMG was increased in older adults vs. young adults. Background EMG activity has been shown to reflect spinal rather than cortical mechanisms while undergoing TMS (Kiers et al., 1993). Although pre- and post-background EMG activity was below the threshold used to determine EMG silence (<10 μV) (Clark et al., 2004), the main effects of age remained. The fact that resting background EMG was higher for older adults than young adults indicates an increase in drive-to-spinal mechanisms rather than cortical mechanisms for motor inhibition (Kiers et al., 1993; Clark et al., 2004). This might be due to the atrophy of white and gray matter in the motor cortical regions, atrophy of the cerebellum, and alterations of the basal ganglia pathways in older adults (Dempster, 1992; Rodríguez-Aranda and Sundet, 2006). Since background EMG activity was higher for older adults than young adults, this supports the theory that older adults have more “noise” in their peripheral motor system. These additional activations are often interpreted as reflecting compensation, but several examples of greater activation are associated with poorer performance in older adults (Gazzaley et al., 2005; Reuter-Lorenz and Lustig, 2005).

There were several limitations to this study that need to be considered. First, the sample consisted of a mix of past music experiences in our non-musician groups. Young adult non-musicians had 0–3 years of training, while older adult musicians had 0–7 years of music training. Various studies have shown that non-musicians with short-term training (i.e., 1-3 years) in early childhood have some music training-related neuroplasticity (Merrett et al., 2013). Our musician group included a mix of professional and amateur musicians. Studies have shown differences regarding executive function in both populations (Hanna-Pladdy and MacKay, 2011; Hanna-Pladdy and Gajewski, 2012). Furthermore, with fewer than 20 participants in each group, the small sample size may have affected the power to detect small effect sizes. Future directions would be necessary to test a controlled sample of musicians (professional and amateur separated) and non-musicians (no formal music training experience), analyzing the type of instrument and type of music training, genetics, and individual differences (Merrett et al., 2013).

## Conclusions

In conclusion, our hypotheses were partially correct. We did not observe significant differences between young and older musicians and non-musicians in resting single-pulse MEP. There was a main effect of age on resting SICI MEP. Older adults have lower rest SICI MEP than young adults. Age and group (musician vs. non-musician) mainly affected the percentage of inhibition at rest. Age also had a main effect on the percentage of inhibition at rest. Older adults (36%) have a greater percentage of inhibition than young adults (16%). However, when controlling for background EMG activity, there was a main effect of the group with musicians (regardless of age). Musicians have lower inhibitions than non-musicians. Overall, despite the greater use of spinal mechanisms, decreased SICI, and increased inhibition percentage in older adults, the results suggest that, during rest, motor circuitry remains intact and functional. However, daily life requires movement. Whether results will change while moving remains to be observed. Regardless, this is the first study to examine musicians and non-musicians across the lifespan using single-pulse and SICI MEP TMS. Future studies will examine single-pulse and SICI MEP during movement across the lifespan and in musicians.

## Data availability statement

The raw data supporting the conclusions of this article will be made available by the authors, without undue reservation.

## Ethics statement

The studies involving humans were approved by the Iowa State University Institution Review Board. The studies were conducted in accordance with the local legislation and institutional requirements. The participants provided their written informed consent to participate in this study.

## Author contributions

PI and ES made substantial contributions to the concept, design of the study, acquisition of the data, analysis, interpretation of the data, draft, and revision of the article. TM and AZ contributed substantially to the data acquisition, analysis, interpretation of the data, draft, and revision of the article. All authors contributed to the article and approved the submitted version.
